# Characterization of Hazelnut Trees in Open Field Through High-Resolution UAV-Based Imagery and Vegetation Indices

**DOI:** 10.3390/s25010288

**Published:** 2025-01-06

**Authors:** Maurizio Morisio, Emanuela Noris, Chiara Pagliarani, Stefano Pavone, Amedeo Moine, José Doumet, Luca Ardito

**Affiliations:** 1 Department of Control and Computer Engineering (DAUIN), Politecnico di Torino, Corso Duca degli Abruzzi, 24, 10129 Torino, Italy; stefano.pavone@studenti.polito.it (S.P.); jose.doumet@studenti.polito.it (J.D.); luca.ardito@polito.it (L.A.); 2 Institute for Sustainable Plant Protection, National Research Council, (IPSP-CNR), Strada delle Cacce, 73, 10135 Torino, Italy; emanuela.noris@ipsp.cnr.it (E.N.); chiara.pagliarani@ipsp.cnr.it (C.P.); amedeo.moine@ipsp.cnr.it (A.M.)

**Keywords:** aerial photos, UAV, *Corylus avellana*, vegetation indices, non-destructive analyses, phenotyping

## Abstract

The increasing demand for hazelnut kernels is favoring an upsurge in hazelnut cultivation worldwide, but ongoing climate change threatens this crop, affecting yield decreases and subject to uncontrolled pathogen and parasite attacks. Technical advances in precision agriculture are expected to support farmers to more efficiently control the physio-pathological status of crops. Here, we report a straightforward approach to monitoring hazelnut trees in an open field, using aerial multispectral pictures taken by drones. A dataset of 4112 images, each having 2Mpixel resolution per tree and covering RGB, Red Edge, and near-infrared frequencies, was obtained from 185 hazelnut trees located in two different orchards of the Piedmont region (northern Italy). To increase accuracy, and especially to reduce false negatives, the image of each tree was divided into nine quadrants. For each quadrant, nine different vegetation indices (VIs) were computed, and in parallel, each tree quadrant was tagged as “healthy/unhealthy” by visual inspection. Three supervised binary classification algorithms were used to build models capable of predicting the status of the tree quadrant, using the VIs as predictors. Out of the nine VIs considered, only five (GNDVI, GCI, NDREI, NRI, and GI) were good predictors, while NDVI SAVI, RECI, and TCARI were not. Using them, a model accuracy of about 65%, with 13% false negatives was reached in a way that was rather independent of the algorithms, demonstrating that some VIs allow inferring the physio-pathological condition of these trees. These achievements support the use of drone-captured images for performing a rapid, non-destructive physiological characterization of hazelnut trees. This approach offers a sustainable strategy for supporting farmers in their decision-making process during agricultural practices.

## 1. Introduction

According to the International Society for Precision Agriculture (ISPA), precision agriculture consists in a method that “gathers, processes, and analyzes temporal, spatial, and individual data” to support more accurate management decisions useful during agricultural practices. Using different tools such as GPS, sensors, drones or unmanned-aerial vehicles (UAVs), satellite imagery, ground stations, and data analytics, farmers can monitor crop conditions, plant and soil health, and environmental variables at a highly detailed level. This allows further precise interventions, improving crop yield, sustainability, and management efficiency, while minimizing waste, environmental impact, and production costs. At present, farmers primarily rely on on-site inspections of plants by qualified personnel, but more recently, high-resolution imaging conducted with UAVs or drones has started to provide new opportunities in various precision agricultural applications, particularly viticulture [1,2]. Compared to other remote sensing platforms, UAVs offer greater flexibility, adaptability, and accuracy, with the advantages of easy displacement and operation [3,4]. However, multi-rotor UAVs suffer from limited battery duration, a constraint that nonetheless allows the handling of small-to-medium-sized devices. Moreover, as other sensing tools, UAVs are influenced by weather conditions (e.g., rain, snowfall, clouds, wind, and fog), limiting their applicability.

In agriculture, image analysis is commonly performed using the visible and IR spectra. Several approaches to analyze the health status of plants have been adopted, based on neural networks (NNs) or vegetation indices (VIs), with promising results. The majority of VIs are proposed as a numerical synthesis of vegetation features, and are calculated based on spectral characteristics associated with an image pixel [5]. In simple terms, VIs result from the combination of surface reflectance at two or more wavelengths, which highlight a particular feature of the plant canopy. Since VIs mainly emphasize the photosynthetic activity of a crop, they are ubiquitously implemented in remote sensing agricultural applications, providing immediate indications of plant fitness in a specific environmental context. The majority of VIs rely on the inverse relationship between the red and near infrared (NIR) band reflectance associated with green vegetation.

Aerial imaging has been applied (i) to survey forests, which harbor different types of plants but often consist of a prevalent botanical species, such as conifers [6]; and (ii) to monitor cultivated fields, where only one type of plant is grown [7,8], with the intent of developing precision agriculture. Forest monitoring generally aims to identify biotic (pests, diseases) or abiotic (pollution, ice, snow, fire, drought) stressors [9], facing the additional problem of considering different types of trees with diverse canopies and crowns. In this case, individual trees are not the target of analysis, contrary to orchard monitoring. Indeed, VIs combined with a random forest model successfully identified the discoloration of the pine tree’s needles as the first stress response signal [10]. Moreover VIs predicted the sanitary status of forest trees, successfully identifying dead plants [11]; other studies focused on tree height and volume analysis [12,13]. As for orchard monitoring, UAVs were exploited to identify the plant species cultivated in a specific area, with yield monitoring purposes [14,15].

Images can be acquired by different types of sensors, including radars, RGB, or infrared (IR), all ending up in the problems of image segmentation or object identification and classification, requiring the adoption of different machine learning (ML) algorithms and notably, deep learning algorithms based on NNs, particularly for hazelnut fields [16,17,18]. At a smaller scale level, the identification of single trees in an orchard was attempted, facing a semantic segmentation problem [15,19,20]. If the distribution and position of individual trees in an orchard are known, specific parameters, i.e., canopy surface, tree architecture, and plant volume, acting as indirect indicators of plant health and growth rate, can be evaluated through image segmentation and 3D reconstruction; these issues were already considered for different orchard types, namely peach [21], olive [22,23,24], almond [25], apple [26], mango [27], cherry [28], pine [29], hazelnut [30,31,32], and grapevine [33,34].

Remote sensing techniques to assess the health status of individual trees in orchards typically consider water stress conditions, attack by pests, or disease symptomatology [35]. The identification of citrus trees infected by huanglongbing and bacterial canker was reached by applying different VIs, both in laboratory [36] and field conditions [37]. Other studies investigated the possibility of identifying verticillium wilt (VW) in olive trees, finding that crown-temperature-based indices, such as the crop water stress index (CWSI), chlorophyll fluorescence, and carotenoid indices, carotenoid reflectance index 2 (CRI2), and the normalized difference vegetation index (NDVI) were good predictors of early and advanced (e.g., water stress) symptoms caused by VW infection [38,39]. Moreover, apple scab could be detected in apple trees based on leaf wetness [40], while fire blight disease development appeared slightly correlated with normalized different spectral indices (NDSIs), generated from visible–NIR reflectance spectra (e.g., green normalized difference vegetation index, GNDVI, NDVI, and normalized difference red edge, NDRE) [41]. Additionally, different VIs, including NDVI, GNDVI, NDRE, and REGNDVI, were applied to monitor the vigor and health status of peach trees [42]. Lastly, recent studies involving convolutional neural networks (CNNs) attempted to identify *Halyomorpha halys* bugs in orchards [43,44]. Overall, although several VIs have been used for pest and disease identification or for the analysis of the water status of plants, a general ready-to-use framework to transfer the results obtained in a single case—and specifically in one plant—to other individuals is not yet available. Both the complexity of disease diagnosis and the diversity of crop species hamper a direct application of the results to other methods of detecting diseases or abiotic stresses of fruit trees [30].

Extreme weather events, mainly heat waves and drought due to the ongoing climate change are inevitably challenging the agricultural sector, causing serious losses in crop productivity and biodiversity [45,46]. Climate alteration favors the spread and the diversity of pathogens affecting crops [47]. This, combined with the increased severity of the diseases further entail a massive use of chemical treatments in most European countries [48], with inevitable onset of critical environmental issues [49,50]. This condition has deeply modified agricultural practices, searching for novel and more sustainable crop protection strategies. In this projected scenario, a timely and steady monitoring of physiological and pathological conditions of crops, including hazelnut, is fundamental for supporting farmers to preserve future harvests and safeguard food security.

European hazelnut (*Corylus avellana* L.) is a major species of interest for its nutritional value, and its kernels are employed worldwide in the chocolate, confectionery, and bakery industries. This pushed hazelnut cultivation outside its native areas, so that both the total surface planted and the tonnage harvested show a worldwide continuous growth trend from 1961 to 2021 reaching an overall production of 1.07 million metric tons in 2023, with Turkey being the first producer and Italy the second, with 765.000 and 98.670 tons, respectively [51]. European hazelnut trees are particularly sensitive to water deficiency, and under specific climate conditions and in areas with poor precipitation, supplemental irrigation is the only way to ensure plant productivity [52,53]. Additionally, hazelnut suffers from the attack by insects (such as leaf beetles, stink bugs, hazelnut weevils, scale insects, moths, aphids, etc.), mites (eriophyds), fungi (powdery mildew), bacteria (*Xanthomonas* and *Pseudomonas* spps.), viruses (e.g., *Apple mosaic virus*) [54,55,56,57], or other environmental stresses, such as intense UV exposure and temperature shifts [58]. Therefore, introducing regular and standardized monitoring systems to evaluate the physiological and sanitary conditions of hazelnut plants would allow a rational and timely management of the irrigation and protection practices. Moreover, implementing innovative technologies to provide steady and real-time monitoring would support preventive strategies to promptly recognize these issues, guiding agronomic interventions in a more timely and effective manner.

The goal of this work was to build models capable of predicting the sanitary status of a plant starting from the VI scores, calculated using a binary classification approach. Since, to our knowledge, no studies using VIs applied to *C. avellana* plants are available, we compared the performances of different VIs to select those effectively suitable to predict the sanitary status of these plants in open-field conditions. Images from hazelnut orchards across various frequency spectra were collected with drones, simultaneously characterizing the physio-pathological status of individual plants based on visual inspection. Due to the erratic distribution of disease or stress symptoms, possibly linked to the bushy structure of hazelnut trees, images were partitioned and each sub-image was treated as a different datapoint. These data were used to calculate VIs and develop and calibrate ML algorithms useful for an accurate characterization of the plant partition.

This approach allowed us to identify the most suitable VIs and ML tools capable of precisely classifying the target plants. A model accuracy of about 65%, with 13% false negatives, was achieved in a way that was rather independent of the algorithms, demonstrating that the selected VIs can be used to successfully infer the physio-pathological condition of hazelnut trees.

In summary, the novelty of the work presented in this paper lies in the following:

- Applying VIs as predictors of health for hazelnut trees, trees with specific characteristics that put them apart from fruit trees where the VI approach has already been applied;
- Considering portions of trees (and not whole trees) in the analysis, to achieve better classification accuracy, and in particular less false negatives;
- Identifying a subset of VIs (GNDVI, GCI, NDREI, NRI, and GI) as best predictors, while excluding others (NDVI SAVI, RECI, and TCARI), in a literature context where the best VI predictors change in function of the tree considered.

## 2. Materials and Methods

### 2.1. Site Description

**Figure 1:**
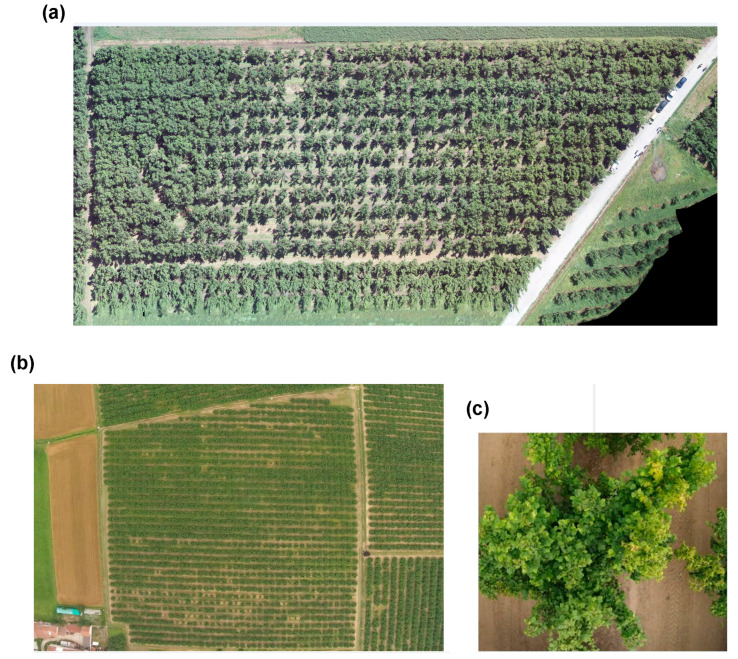
Aerial images of the two hazelnut fields used in this study: (**a**) Farigliano and (**b**) Carrù fields. (**c**) Representative image of a single hazelnut plant in the Carrù field.

This research was conducted during the summer season of 2022 in hazelnut fields located in the Cuneo province in the Piedmont region, Northern Italy. Images were collected from two different orchards. The first field was in Carrù (44°27′40.8″ N 7°49′59.5″ E) with 127 plants of 2 m height, planted at 4 m × 4 m spacing within and between rows, in parallel and contiguous rows on a flat terrain. The second field located in Farigliano (Dogliani, 44°32′37.8″ N 7°54′52.5″ E) hosted 58 plants of 3 m height, planted at the above spacing on a flat surface (Figure 1).

### 2.2. Image Collection

Remote-sensed images for crop telemonitoring were collected using a P4 Multispectral drone (DJI, Shenzhen, China). The drone is equipped by the company with a multispectral camera in the RGB, red edge (RE) (730 nm), and near-infrared (NIR) (840 nm) frequencies, allowing a 2 M pixels resolution per image. For both fields, three flights were conducted at monthly intervals during the plant vegetative season (May–August).

To maximize image resolution, the drone was piloted to capture one image per plant from a 10 m height. Camera settings were defined to obtain a ground sample distance of 1 cm/pixel. Based on detailed maps of the field, a precise drone path was defined, with stops directly above each plant. The same path was repeated during each shooting. The UAV flight was planned in a flight-based automated manner, designed and executed by the DJI GSPro App, using a polygon grid flight plan with 10 m altitude, a velocity of 1.5 m/s, and with the capture mode “Hover & Capture at Point”.

### 2.3. Image Processing

#### 2.3.1. Plant Recognition

**Figure 2:**
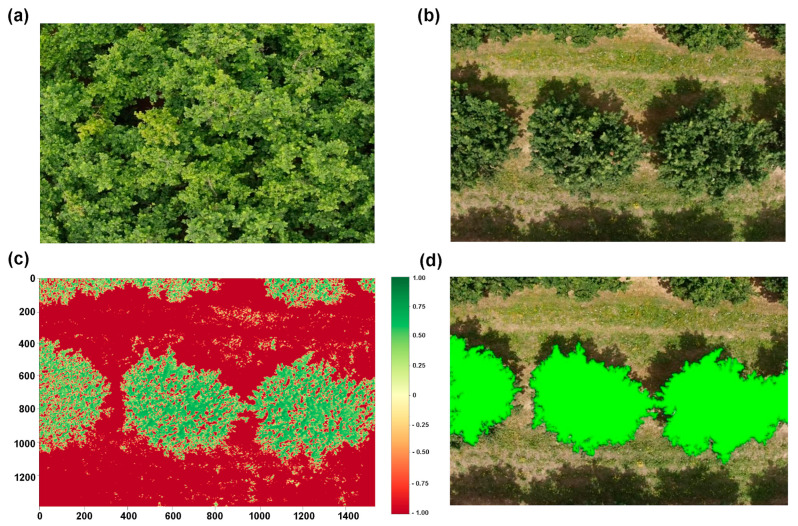
Pre-processing of images for plant recognition and identification of the hazelnut tree canopy. (**a**) Image collected in the Carrù field showing multiple and overlapping canopies; (**b**) RGB image collected in the Farigliano field showing well-separated trees; (**c**) example of application of the normalized difference vegetation index (NDVI) to define the plant contours of the image shown in (**b**) (NDVI values are shown on the key colors); (**d**) the same image of (**b**,**c**) obtained after excluding pixels with NDVI values < 0.2, with plant contours defined and shown in green.

Each photograph was taken from the zenith above each tree. When images did not perfectly correspond to an individual plant, a pre-processing step was performed. For large plants with overlapping canopies (Figure 2a), a geometric cropping approach was adopted, based on the exact position of the trunk and the distance between plants. For instance, based on the 4 × 4 m distance of planting between and within rows, images were cropped to include a 4 × 4 m^2^ area, positioning the trunk in the center of the image.

For young plants with no overlapping canopy (Figure 2b), pixels corresponding to the plant were isolated from background pixels representing soil, using the normalized difference vegetation index (NDVI) (see 2.5 for its definition). Since soil typically has an NDVI value much lower than the plant canopy [4], pixels with NDVI values < 0.2 were excluded from subsequent analyses. From an RGB plant image (Figure 2c), the corresponding NDVI values are shown, with soil pixels below the threshold highlighted in red. After applying a hierarchical contouring algorithm and removing smaller contours contained within larger ones, plant contours were finally identified (green areas in Figure 2d).

#### 2.3.2. Image Slicing

**Figure 3:**
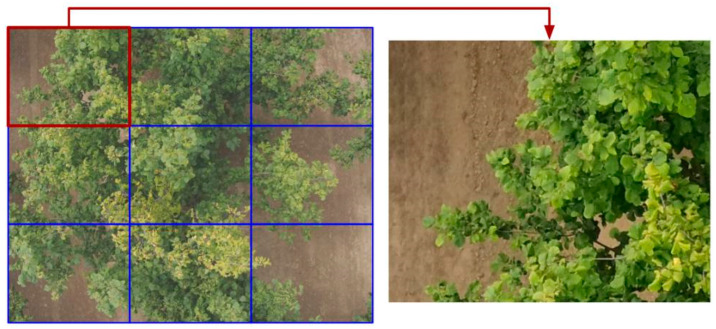
Example of plant image slicing. The inset represents a magnification of the slice bordered in red. Each slice was visually inspected, and binary classified as healthy/unhealthy.

Initially, we intended to classify each plant as healthy/unhealthy. However, symptoms of abiotic stress or pathogen attack (e.g., downward curling of the leaf lamina, leaf yellowing or reddening, leaf scorch, wilting of some portions of the canopy and/or branch collapse) were visible only in a portion of the plant in around half of the collected images. Additionally, as VIs are computed per pixel and averaged to compute a value per image, this would likely produce a high number of false negatives. Therefore, images were geometrically partitioned into nine individual pictures (Figure 3), each representing a surface slightly larger than 1 m^2^, so that VIs were associated with each segmented image.

### 2.4. Tree Tagging

Each sliced image was visually inspected and classified in binary terms, i.e., healthy vs. unhealthy. To increase accuracy, the classification was made independently by three different persons with more than 10 years of botanic experience. In case of disagreement, the team met and discussed until an agreement was reached.

Binary classification in two classes only (healthy, not healthy) is limited, and ideally each image could be broadly characterized specifying the level of pathological status, with multiple possible values. However, due to the nearly limitless physio-pathological conditions of a plant and considering the reduced number of images collected, a binary classification was adopted.

### 2.5. Vegetation Indices (VIs)

Vegetation indexes are numerical indicators computed from the different spectral ranges of an image, with the intent of characterizing vegetation properties [5]. VIs are computed per pixel, so they have to be aggregated to provide a VI per image: the average value of all pixels of an image is computed and the VI of the image is considered. Due to the image slicing strategy adopted, VIs are associated with each sub-image.

Nine different VIs were tested, based on (i) vigor indices, used to differentiate plants from their surrounding (ground, soil, roads, people, etc.) and (ii) chlorophyll indices, suitable for evaluating the plant physiological conditions (e.g., the intensity of the green color of leaves as a proxy of the chlorophyll content, nitrogen reflectance, etc.). The VIs used in this work are listed below.

(1) NDVI, one of the most used VIs in remote sensing measurements, quantifies vegetation by measuring the normalized difference between NIR bands, strongly reflected by vegetation, and red (RED) bands. NDVI is calculated pixel-to-pixel as follows:

(1) $$NDVI=\frac{NIR-RED}{NIR+RED}$$

NDVI can range from −1 and +1, with positive values attesting the presence of vegetation, strictly negative values representing water (or clouds for satellite imagery), and values around 0 indicating soil properties (dirt, rock, sand, etc.). Values > 0.3 characterize green areas such as crops or forests. Consequently, NDVI below a certain threshold, due to a low reflectance of the NIR bands, can be indicative of plants undergoing stress events.

(2) GNDVI, a proxy of the plant photosynthetic activity, provides information on the nitrogen and water content of plants. It employs the NIR and green bands (GREEN) but, contrary to NDVI, it is more sensitive to changes in chlorophyll content. For this, GNDVI is expected to more accurately determine the physiological status of plants and has a higher saturation point. Compared to NDVI, this chlorophyll-based index is normally applied at later stages of plant growth, as it saturates later than NDVI. Therefore, while NDVI is more suitable to estimate crop vigor during the early growing stages, GNDVI can be used in crops with dense canopies or in more advanced stages of development. Like NDVI, GNDVI can range from −1 to +1 and is computed as follows:

(2) $$GNDVI=\frac{NIR-GREEN}{NIR+GREEN}$$

(3) The green chlorophyll vegetation index (GCI) is used to assess the sanitary condition of a crop, based on its chlorophyll content. GCI is derived from the NIR and GREEN bands and is calculated using the following formula, assuming values from −1 to +infinity:

(3) $$GCI=\frac{NIR}{GREEN}-1$$

(4) NDREI is commonly adopted to determine the concentration of chlorophyll in plants, mainly in the mid-to-late growing season when the plant fruits start to be mature. NDREI is based on the red-edge (RE) band measurement and is calculated using the following formula:

(4) $$NDREI=\frac{NIR-RED\_EDGE}{NIR+RED\_EDGE}$$

(5) The red-edge chlorophyll index (RECI) assesses the chlorophyll concentration in leaves and, as NDREI, is determined using the ratio of reflectivity in the NIR and RE bands:

(5) $$RECI=\frac{NIR}{RED\_EDGE}-1$$

(6) The nitrogen reflectance index (NRI) aims at determining the nitrogen content of plants, an indispensable macronutrient present in proteins, enzymes, and chlorophyll, whose deficiency causes stunted growth, small leaves with pale green or yellowish color, and lower chlorophyll concentration. Since low NRI values are commonly associated with higher reflectance, healthy plants are expected to exhibit lower NRI values.

(6) $$NRI=\frac{GREEN-RED}{GREEN+RED}$$

(7) The greenness index (GI) directly correlates with the chlorophyll content and therefore plant’s health status; as for NRI, low GI values apply to healthy crops

(7) $$GI=\frac{GREEN}{RED}$$

(8) The transformed chlorophyll absorption and reflectance index (TCARI) allows the identification of chlorotic areas in a field, due to nutritional deficiencies or plant diseases.

(8) $$TCARI=3\;\left(RED\_EDGE-RED\right)\;\frac{0.2\;(RED-GREEN)}{RED\_EDGE-RED}$$

(9) The soil-adjusted vegetation index (SAVI) applies a correction to the NDVI to reduce the influence of soil brightness in areas where vegetation coverage is scarce. It is calculated with the following formula, where L indicates the correction factor.

(9) $$SAVI=(1+L)\;\frac{NIR-RED}{NIR+RED+L}$$

L can range from 0 to 1 and it is set to 1 for areas with scarce vegetation, but as it can assume different values depending on the environment, it is frequently set to 0.5. Noteworthy, for L = 0, the SAVI value corresponds to NDVI.

### 2.6. Machine Learning Protocols

Three classical supervised ML algorithms, i.e., random forest, K-nearest neighbors (KNN), and logistic regression (LR) were applied and compared for classification [59]. In all cases, models were built using Python 3.7, with the libraries Tensorflow 2.11, scikitlearn 1.2, and PyTorch.1.13, adopting random search to optimize the hyperparameters and selecting k-fold = 5.

For random forests, the selected hyperparameters are #estimators = 100, criterion = Gini, no max depth. For KNN, the hyperparameters selected are neighbors = 5, uniform weights, ball tree computation of distance with leaf size = 30. For LR, the hyperparameters selected are lbgs solver, penalty l2.

## 3. Results

### 3.1. Image Processing and Binary Classification of Plants

**Figure 4:**
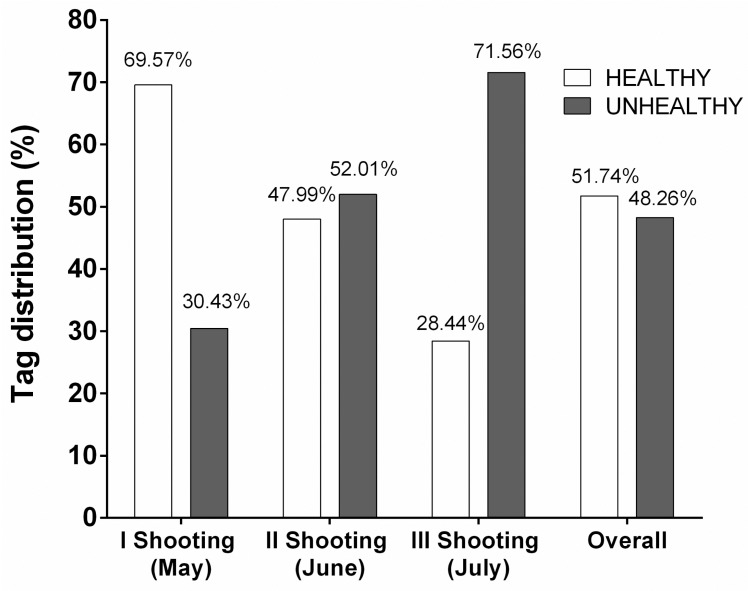
Distribution of the whole dataset of images collected from hazelnut plants following binary classification in terms of “healthy/unhealthy”, across the whole acquisition period from May to July (three shooting time points).

After image segmentation, a total of 4995 pictures were obtained during the whole season (from May to July). Image portions containing mostly ground soil were excluded, leading to a final dataset consisting of 4112 images. The class distribution of the whole dataset after visual inspection and binary classification across the whole acquisition period is shown in Figure 4. A clear increase in the number of images classified as “unhealthy” occurred along the season, with a ratio of healthy vs. unhealthy of 2.28, 0.92, and 0.39 in the three shooting time points, respectively. Considering the whole dataset, the two classes were evenly distributed.

### 3.2. Computation and Selection of VIs

**Figure 5:**
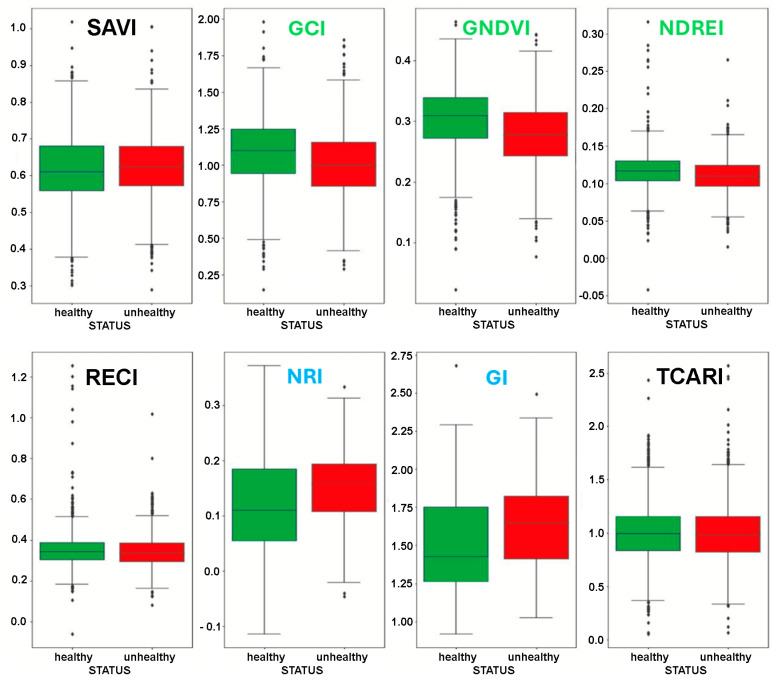
Boxplots of the vegetation indices calculated on the image dataset.

The mean VIs were calculated for all pixels of an image, and this value was taken as the VI of the whole image. Figure 5 shows the boxplots for the VIs computed over the whole dataset of images, tagged as ‘healthy’ and ‘unhealthy’ classes.

The class distribution of “healthy/unhealthy” plants appeared for the SAVI (which basically includes NDVI), RECI, and TCARI VIs. Indeed, SAVI mainly discriminates vegetation canopies from the ground. Both RECI and TCARI include red edge, suggesting that the content of this information is not highly meaningful for this study. Therefore, SAVI, RECI, and TCARI were excluded from further analyses.

Conversely, the GCI, GNVI, and NDREI metrics provided a general trend showing higher and lower values for healthy and unhealthy plants, respectively. Additionally, higher NRI and GI values were calculated for unhealthy plants vs. healthy plants, as expected.

### 3.3. VIs as Predictors of Health Status

The dataset was divided into a training set and a testing set in the proportions of 80–20%, using stratified random sampling. The best combinations of hyperparameters were achieved through parameter optimization methods, such as random search and grid search. Moreover, for each model, a k-fold cross-validation process was used and the evaluation was made showing confusion matrices and measures of accuracy, precision, recall, and F1-score.

**Figure 6:**
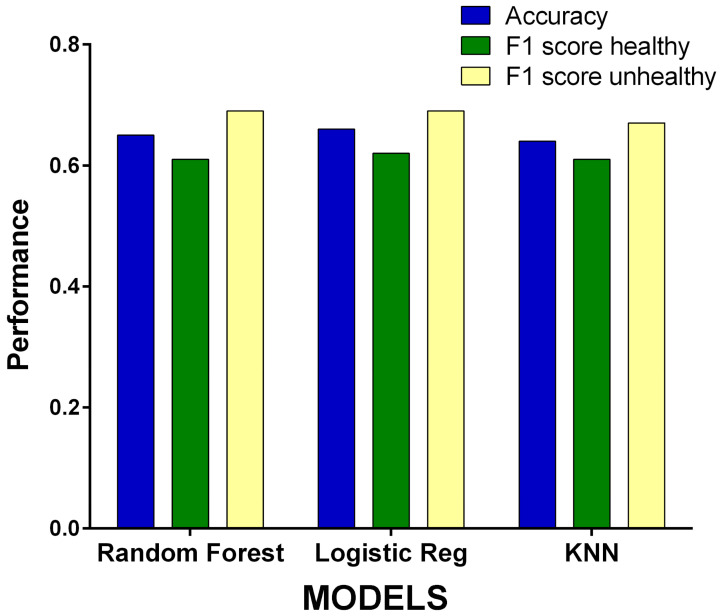
Performances of the different supervised machine learning algorithms applied to the selected vegetation indices GNDVI, GCI, NDREI, NRI, and GI. The performance is expressed in terms of accuracy and F1-score.

The results of the computation of the above selected VIs, i.e., GNDVI, GCI, NDREI, NRI, GI, and their analyses with different ML algorithms are shown in Figure 6 and Table 1. Overall, the three different ML approaches adopted produced similar results. The LR method performed best with an accuracy of 0.66 and F1-scores of 0.62 and 0.69 for “healthy” and “unhealthy” plants, respectively. Random forest scored second in terms of accuracy, with 0.65, and obtained F1-scores of 0.61 and 0.69, while the KNN method achieved a slightly lower level of accuracy of 0.64 with F1-scores of 0.61 and 0.67.

In agriculture, the most important issue is to recognize unhealthy plants, and false negatives represent the most dangerous failure of a diagnostic technique. In this regard, the KNN identified 132 false negatives (out of 823 data points, 16%), while only 112 and 113 false negatives were computed by the random forests or the LR method, corresponding to about 13% of cases.

## 4. Discussion

In this work, we used images acquired with drones, having a resolution up to 16 times higher than non-military satellites, such as Landsat 8 which produces images with a resolution of 15–100 m per pixel (based on the band considered), unsuitable for individual tree monitoring.

To evaluate whether VIs could successfully predict the sanitary condition of a hazelnut grove, multispectral and hyperspectral sensors in the visible and IR range were considered. Among the nine VIs considered, SAVI, RECI, and TCARI could not successfully discriminate between healthy and unhealthy plants. Since SAVI is based on NDVI, we assumed that NDVI was also unsuitable for such discrimination analyses. On the contrary, GNDVI, GCI, NDREI, NRI, and GI were used to build models capable of correctly classifying plants, achieving an average accuracy of 65%. The significance of these results did not statistically differ when different methods were applied, such as random forest, LR, or KNN. Although a similar approach was already adopted for other crops [10,11,36,37,38,39,41,42], to our knowledge, no reports concerning hazelnut trees are available. This possibly results from the phenotypic traits of these plants, having a bushy pattern with large leaves and dense foliage, making it difficult to treat them as fruit trees. Such features could also explain the reduced performances of NDVI obtained in this work.

A wide range of accuracy is reported in the literature when the identification of the sanitary status of orchard trees using different VIs is evaluated. This variability is linked to different factors, including the kind of orchard and the size and age of the trees, the stress condition considered, and the time of detection. For example, the WI (water index), ARI, and TCARI1 VIs achieved 92% accuracy in the identification of canker disease in the fruits of citrus trees [36], but only when the orchard was evaluated at late stages of infection (i.e., with clearly visible symptoms), while the accuracy dropped to around 46% at early stages of disease development. In another work, NIR_R scored as the best predictor for citrus tree and canker disease classification analysis, while NDVI, GNDVI, SAVI were inefficient [37]. In this case, a classification accuracy ranging between 67 and 85% was reported, but with false negatives rates varying between 7 and 32%, possibly due to the low image resolution (4.5 cm per pixel) or to the fact that trees were classified as “with. /without disease” as a whole, without considering possible erratic or localized distribution of symptoms. In addition, the performances of a number of VIs (including NDVI and RDVI) were also tested to discriminate the level of severity of the disease, but in this case, the results of their classification were not reported [38]. In a study conducted on apple trees affected by the fire blight disease, NDVI, GNDVI, NDRE showed classification accuracies between 74 and 90%, with false negatives in the range of 3–11% [41], but the number of plants considered was very limited. Moreover, the NDVI, GNDVI, NDRE, REGNDVI values of peach trees were computed, assuming that higher values identify the most vigorous trees [42]; however, in this case, plant vigor was not correlated with any patho-physiological analysis of the trees. The accuracy results of this study are in line with the studies reported above, but not better. On one hand, a straight comparison is difficult to do, because, while the usage of VIs is similar, the object of study (hazelnuts) is different. Differences in accuracy results could depend either on the intrinsic characteristic of hazelnuts (foliage, bushy structure) or on the specificity of the research goal (quite broad in this study, in terms of healthy/not healthy, but more specific in other studies, targeting, for instance, only canker disease).

In other works, trees were generally considered as a whole and classification was made in terms of “healthy/unhealthy plant”. The image splitting strategy adopted herein prompted us to individually tag each sub-image and compute the selected VIs as the average of each pixel in the image. This approach provided a higher precision in the characterization of the phenotypic traits of plants, likely associated with the effects of abiotic and/or biotic stressors. This strategy was particularly suitable considering the typical hazelnut tree architecture, a bush composed of different independent branches possibly displaying different and erratic symptoms.

Moreover, this splitting strategy allowed us to minimize the risk of false negatives detection. Indeed, false negative predictions are a dangerous issue when the early monitoring of biotic or abiotic stress conditions is pursued. In fact, in 10% of the trees considered, only one of the nine sub-images was classified as “unhealthy”, thus contributing to limiting the number of false negatives and simultaneously providing a more correct classification of the tree. However, despite this approach, up to 13% of the plants were predicted as “unhealthy” and could therefore be incorrectly classified; nonetheless, such value is similar or below the false negative values reported previously (e.g., 7–32% in the study by [37] and 3–11% in [41].

Overall, these results lay the foundations to develop a UAV-based monitoring service suitable to flag potentially stressed or diseased plants on a whole field surface, thereby enabling farmers to rapidly identify the plants requiring prompt agronomical intervention. In this scenario, a drone-based monitoring service capable of surveying large fields, at high frequency (e.g., at least once a week) with repeated inspections of flagged plants, can be envisaged. This approach would help farmers to save time and money, thanks to the more frequent and deeper controls of flagged individuals in a timely manner.

The accuracy of plant classification using the proposed approach would benefit from the use of larger datasets, of other computational techniques of image analysis, such as neural networks, or from the use of a wider frequency of the spectrum considered. In addition, a more detailed definition of the plant parameters, rather than the binary classification adopted herein, would strongly increase the value of this approach. However, given the almost infinite kind of stresses occurring on plants, even simultaneously, a pragmatic approach should be adopted, focusing on defining a finite number of patho-physiological conditions specific for the crop of interest, e.g., “water stress”, “pest attack”, “pathogen infection”, or “no symptoms”.

## 5. Conclusions

In this work, we tested the suitability of different VIs to precisely classify the sanitary status of hazelnut plants, adopting in parallel a binary classification of trees based on visual inspection. Overall, the novelty of this work lies in the possibility of applying VIs as predictors of sanitary conditions in this crop, characterized by specific plant architecture and canopy morphology traits. Out of nine different VIs tested, GNDVI, GCI, NDREI, NRI, and GI were selected as the best predictors, contrary to NDVI, SAVI, RECI, and TCARI, indicating that the suitability of VIs changes according to the crop considered. Moreover, the specific features of hazelnut trees asked for the adoption of image segmentation strategies to increase the classification accuracy and reduce false negatives. Overall, a model accuracy of about 65%, with 13% false negatives was achieved in a way rather independent of the algorithms, demonstrating that the aforementioned VIs can be used to successfully infer the physio-pathological condition of hazelnut trees.

Conclusively, we gathered and analyzed data related to the physio-pathological status of cultivated hazelnut plants to preserve the qualitative and quantitative traits of this crop, including product yield, ultimately aiming at reducing agricultural management costs (e.g., irrigation and pest control measures). Computing VIs from images collected with a small commercial drone coupled to a multispectral camera allowed a prompt identification of unhealthy hazelnut plants, at early times, providing farmers with an accurate tool to identify trees with specific physio-pathological issues. A frequent, continuous, large-scale, and real-time monitoring of the physio-pathological condition of plants, currently mostly relying on human intervention, can optimize agronomic practices in the medium- to long-term perspective, resulting in cost reductions and improvements in productivity and the quality of the product. We believe that the proposed approach can be extended to the entire fruit sector.

## Figures and Tables

**Table 1:** Performance of the different machine learning classification algorithms used in this work, calculated on the test sets and expressed as figures of merit (precision, recall, F1-score).

Tested Model	Binary Classification	Precision	Recall	F1-Score
Random forest	0	0.67	0.56	0.61
	1	0.64	0.74	0.69
	Accuracy			0.65
	Macro average	0.66	0.65	0.65
	Weighted average	0.66	0.65	0.65
Logistic regression	0	0.67	0.57	0.62
	1	0.65	0.73	0.69
	Accuracy			0.66
	Macro average	0.66	0.65	0.65
	Weighted average	0.66	0.66	0.65
KNN	0	0.64	0.58	0.61
	1	0.64	0.70	0.67
	Accuracy			0.64
	Macro average	0.64	0.64	0.64
	Weighted average	0.64	0.64	0.64

Note: The test set included 823 images, of which 397 were classified as “Healthy” and 426 as “Unhealthy”.

## Data Availability

The raw data supporting the conclusions of this article will be made available by the authors upon request.
